# Exploring the connections between psychiatric disorders and climate change

**DOI:** 10.1192/j.eurpsy.2024.978

**Published:** 2024-08-27

**Authors:** I. Marinić, L. Mužinić Marinić

**Affiliations:** Clinic for Psychiatry, University Hospital Dubrava, Zagreb, Croatia

## Abstract

**Introduction:**

Considering the increased occurrence of climate changes in the world and their consequences on human health and quality of life, there is an increase in psychiatric disorders, including anxiety disorders, mood disorders, and stress related disorders caused by climate changes.

**Objectives:**

To explore the connections between psychiatric disorders and certain types of climate change.

**Methods:**

Data from research related to climate change and its impact on mental health are presented.

**Results:**

Research indicates an increase in psychological disorders related to climate change from several diagnostic categories, consequently to the acute and long-term effects of climate changes, depending on the type of climate event, individual sensitivity, socioeconomic conditions, community support and assistance, and response to therapeutic interventions.

**Conclusions:**

In addition to raising awareness of the impact of climate change on psychological health, it is important to develop strategies for providing psychological and psychiatric assistance, both immediately after a climate event and during long-term exposure to adverse climate conditions, especially for vulnerable groups.

**Disclosure of Interest:**

None Declared

